# Prevalence of Fabry disease-causing variants in the UK Biobank

**DOI:** 10.1136/jmg-2022-108523

**Published:** 2022-08-17

**Authors:** Mark Gilchrist, Francesco Casanova, Jess S Tyrrell, Stuart Cannon, Andrew R Wood, Nicole Fife, Katherine Young, Richard A Oram, Michael N Weedon

**Affiliations:** 1 College of Medicine and Health, University of Exeter Medical School, Exeter, UK; 2 College of Medicine and Health, University of Exeter, Exeter, UK; 3 Genetics of Complex Traits, University of Exeter Medical School, Exeter, UK; 4 Institute of Biomedical and Clinical Science, University of Exeter Medical School, Exeter, UK

**Keywords:** Screening, Cardiovascular Diseases, Genetics, Nephrology

## Abstract

**Background:**

Fabry disease is an X-linked lysosomal storage disorder resulting from deficiency of the alpha-galactosidase A enzyme leading to accumulation of globotriaosylceramide in multiple organ sites with prominent cardiovascular and renal involvement. Global prevalence estimates of Fabry disease based on clinical ascertainment range from 1 in 40 000 to 1 in 170 000. We aimed to determine the prevalence of Fabry disease-causing variants in UK Biobank.

**Methods:**

We sought *GLA* gene variants in exome sequencing data from 200 643 individuals from UK Biobank. We used ACMG/AMP guidelines (American College of Medical Genetics/Association for Molecular Pathology) to classify pathogenicity and compared baseline biomarker data, hospital ICD-10 (International Classification of Diseases version-10) codes, general practitioner records and self-reported health data with those without pathogenic variants.

**Results:**

We identified 81 *GLA* coding variants. We identified eight likely pathogenic variants on the basis of being rare (<1/10 000 individuals) and either previously reported to cause Fabry disease, or being protein-truncating variants. Thirty-six individuals carried one of these variants. In the UK Biobank, the prevalence of likely pathogenic Fabry disease-causing variants is 1/5732 for late-onset disease-causing variants and 1/200 643 for variants causing classic Fabry disease.

**Conclusion:**

Fabry disease-causing *GLA* variants are more prevalent in an unselected population sample than the reported prevalence of Fabry disease. These are overwhelmingly variants associated with later onset. It is possible the prevalence of later-onset Fabry disease exceeds current estimates.

**WHAT IS ALREADY KNOWN ON THIS TOPIC**

- Fabry disease is typically thought of as a rare X-linked disease with a prevalence between 1 in 40 000 and 1 in 140 000. Newborn screening suggests a higher prevalence but relies on dried blood spot testing which is unreliable in affected females.

**WHAT THIS STUDY ADDS**

- This is one of the largest Fabry screening efforts ever undertaken and to our knowledge the largest based on exome sequencing and the largest in an unselected adult population including men and women. The prevalence of Fabry disease-causing variants is 1 in 5573 older adults. These are predominantly associated with late-onset disease or may demonstrate incomplete penetrance.

**HOW THIS STUDY MIGHT AFFECT RESEARCH, PRACTICE OR POLICY**

- It is not known how to manage infants identified through newborn screening efforts. Our data offer significant insight into the likely clinical course of children identified with these late-onset variants which can inform clinical monitoring and treatment guidelines.

## Introduction

Fabry disease (OMIM 301500) is an X-linked lysosomal storage disease caused by variants in the *GLA* gene. *GLA* encodes the alpha-galactosidase A (α-Gal A) enzyme responsible for degradation of glycosphingolipids, notably globotriaosylceramide and globotriaosylsphingosine.^1^ Deficient activity of α-Gal A leads to progressive accumulation of these substrates over time in every organ. There is considerable heterogeneity in clinical presentation in terms of pattern of organ involvement and age of onset between variants and even within families with the same variant.

Though X-linked, Fabry disease affects females through random X-inactivation. The pattern of organ involvement and severity consequently can be comparable with that of classically affected males.^2^ Prior to the advent of enzyme-replacement therapy, life expectancy in female ‘carriers’ of Fabry disease-causing variants was 15 years less than the general population.^3^

Global prevalence estimates of Fabry disease based on clinical ascertainment range from 1 in 40 000 to 1 in 170 000.^4^ Newborn screening studies examining α-Gal A activity on dried blood spots with subsequent confirmatory genetic testing have suggested a higher prevalence of Fabry disease, particularly variants associated with late-onset Fabry disease, than might be expected from studies of confirmed Fabry cases. Spada *et al* found a prevalence of known disease-causing pathogenic variants of 1 in 4600 in consecutively screened newborn Italian boys.^5^ The prevalence of late onset to classic phenotype-associated pathogenic variants in this cohort was 7:1. Sawada *et al* screened 599 711 male and female newborns in western Japan and detected a prevalence of pathogenic variants of 1 in 11 854 following initial screening by measuring α-Gal A activity on dried blood spot tests.^6^ A US-based study arrived at a prevalence of 1 in 21 973.^7^ The highest suggested prevalence was from a study of 171 977 Taiwanese newborns which arrived at a prevalence of approximately 1 in 1250. Much of this high prevalence was driven by a single variant, c.936+919G>A (IVS4+919G>A). This deep intronic variant accounted for 86% of those identified.^8^ While it will not be known for many years what proportion of those individuals with pathogenic variants go on to develop clinical manifestations of Fabry disease, it is worth noting that 3 of the 57 individuals followed up as a result of Sawada *et al*’s study had developed manifestations of Fabry disease by the age of 5 years and commenced enzyme-replacement therapy by the age of 10 years.^6^

When assessed in cohorts referred for testing with a high clinical suspicion of Fabry disease (positive family history and/or characteristic phenotype), α-Gal A activity in dried blood spot tests has a sensitivity reported to be 100% in males, though this is halved in females.^9^ Sensitivity in lower-risk cohorts is not known. Efforts to identify patients with early manifestations based on a single pathological finding, such as early-onset stroke, reduced estimated glomerular filtration rate (eGFR) or the need for renal-replacement therapy, have notoriously low yields.^10–13^

The UK Biobank offers an unparalleled opportunity to determine the prevalence of Fabry disease-causing pathogenic variants in an unselected adult population. Unselected exome screening for Fabry disease-causing pathogenic variants has not been performed at this scale previously.

Using the UK Biobank, we sought to describe the prevalence of known Fabry disease-causing variants and associated phenotype. Further, we aimed to determine whether participants with characteristic clinical manifestations of later-onset Fabry disease would be predictive of the presence of known Fabry disease-causing variants.

### Methods

The UK Biobank recruited a sample of more than 500 000 individuals between the ages of 37 and 73 years from 2006 to 2010 across the UK.^14^ At baseline, participants completed detailed questionnaires on health, sociodemographic and lifestyle factors. Blood and urine samples were obtained for genetic analysis along with a broad range of markers of health and disease.

Based on literature review, we created two cohorts of individuals with combinations of comorbidity which would be suggestive of a potential underlying diagnosis of Fabry disease using Hospital Episodes Statistics (HES) data. The first was men under the age of 60 years with chronic kidney disease (defined as eGFR <60 mL/min or albumin–creatinine ratio (ACR) >2.5 mg/mmol) and cardiac disease (including ischaemic heart disease, cardiomyopathy, dysrhythmias or cardiac failure) and hearing loss. The second group was men under the age of 60 years with chronic kidney disease (defined as eGFR <60 mL/min or ACR >2.5 mg/mmol) and cardiac disease and cerebrovascular disease. For a full list of HES codes used, please see online supplemental file.

10.1136/jmg-2022-108523.supp1 Supplementary data

Exome sequencing data from 200 643 individuals from the UK Biobank were examined for variants in the *GLA* gene. Likely pathogenic variants were searched for in ClinVar and Human Gene Mutation Database with subsequent review of supporting literature.

Variants are annotated by AlaMut batch software V.1.8 (Interactive Biosoftware, France) using the NM_000169.2 transcript for *GLA*. The pathogenic variants can be missense or protein truncating. We defined a protein-truncating variant (PTV) as a variant that is predicted to cause a stop gain, a frameshift or abolish a canonical splice site (−2 or +2 bp from exon). In this study, we excluded PTVs in the last exon of each gene.

We reviewed all heterozygous missense and PTV in UK Biobank that were observed at minor allele frequency <0.001 in gnomAD V.2 (N=141 456).^15^ ACMG/AMP (American College of Medical Genetics/Association for Molecular Pathology)guidelines were used to classify missense variants as pathogenic/likely pathogenic.^16^ All PTVs were classified as pathogenic.

Pathogenic variants (missense and PTVs) were reviewed in Integrative Genomics Viewer to remove false positive variants. The variants considered to be excellent quality^17^ and deemed to be high confidence by LOFTEE^15^ (only for PTV) were included in the analysis.

Data were analysed using Stata V.16 (Statacorp, Texas, USA). Comparisons between the group with Fabry-causing variants and the remainder of the Biobank cohort with exome sequencing data were performed using Fisher’s exact test.

HES data for each individual were examined for possible Fabry-associated phenotypes mirroring the risk cohort search. In addition, primary care data were searched for the terms associated with classic as well as late-onset Fabry phenotypes (see online supplemental data).

10.1136/jmg-2022-108523.supp2 Supplementary data

Self-reported data for cardiovascular disease, hearing loss and family history of cardiovascular disease from questionnaires at the assessment centre were compared in those with and without Fabry disease-causing variants.

Kidney pathology was assessed by CKD-EPI eGFR and urinary ACR.

## Results

### Pathogenic variant prevalence

We identified 81 coding variants in the *GLA* gene. Eight of these variants were rare (<1 in 10 000 individuals) and either previously reported to cause Fabry disease or PTVs (table 1). Thirty-six individuals, (15 male, 21 female) carried one of these *GLA* variants (see table 2 for variant details). Thirty-three of the 36 individuals were recorded as being of European ancestry. The cardiac predominant phenotype causing c.644A>G (p.N215S) variant (18 of 36) was the most common variant. Six of 36 had the c.1087C>T (R363C) variant, 4 of 36 had c.1067G>A (p.R356Q). The c.335G>A (p.R112H), c.902G>A (p.R301Q) and c.593T>C (p.I198T) variants were each identified in two individuals. There were single individuals with c.695T>C (p.I232T) and c.718_719del (p.K240fs) variants.

**Table 1:** Disease-causing variants in the UK Biobank with associated disease types and reported ranges of enzyme activity from clinical populations and in vitro studies

Variant	Protein change	N in biobank	gnomAD freq (MAF)	Exon	Disease type	Enzyme activity
						(% WT)
c.644A>G	p.N215S	18 (1/11 147)	1/183 422	5	Late onset	5–39.5 (18–21)
c.1087C>T	p.R363C	6 (1/33 461)	2/183 376	7	Late onset	7.5 (21)
c.1067G>A	p.R356Q	4 (1/50 161)	2/183 292	7	Late onset	12.9–86.9 (8, 19–22)
c.335G>A	p.R112H	2 (1/100 322)	3/183 240	2	Late onset	<5 (19)
c.902G>A	p.R301Q	2 (1/100 322)	Not seen	6	Both described	3–5.6 (20–22)
c.593T>C	p.I198T	2 (1/100 322)	2/18 1907	4	Late onset	10–64.7 (20, 21, 25)
c.718_719del	p.K240fs	1 (1/200 643)	Not seen	5	Classic	0.5–2.5 (24, 26)
c.695T>C	p.I232T	1 (1/200 643)	Not seen	5	Late onset	7.2–15 (19–22, 27)

MAF, minor allele frequency; WT, wild type.

**Table 2:** Baseline characteristics of participants with Fabry disease-causing variants

	Combined	Male (M)	Female (F)	Background*	Significance
n (%)	36 (100)	15 (42)	21 (58)	44.9 M, 55.1 F	p=0.69†
Age (years), mean (SD)	54.8±8.4	53.1±8.7	55.3±8.4	56.7±8.2M, 56.3±8.0 F	p=0.13 M, p=0.60 F‡
Diagnosed Fabry disease	3	2	1	0	NT
Cardiovascular disease, n (%)	10 (27.8)	6 (40)	4 (19)	18 221 (20) M, 11 557 (10) F	p=0.099 M, p=0.27 F
Hearing impairment, n (%)	1 (2.8)	1	0	3773 (1.88)	NT
CKD-EPI eGFR mL/min/1.73 m^2^§	90±15	93±14	89±15	92±14 M, 91±13 F	p=0.91 M, p=0.50 F‡
Microalbuminuria (n=35)¶, n (%)	7 (20)	5 (35.7)	2 (9.5)	4935 (5.6) M, 5185 (4.9) F	p=0.0007 M, p=0.27 F

P values are for combined men and women with Fabry-causing variants versus the background cohort unless specified otherwise using Fisher’s exact test.

*n=200 596 (194 578 for ACR data).

†Pearson’s χ2.

‡Independent samples t-test.

§Creatinine missing for one woman.

¶ACR missing for one man.

**

ACR, albumin–creatinine ratio; CKD-EPI eGFR, Chronic Kidney Disease Epidemiology Collaboration estimated glomerular filtration rate; NT, not tested.

In the UK Biobank, the overall prevalence of Fabry disease-causing variants is 1 in 5573. Only one variant, c.718_719del (p.K240fs), is consistently associated with the classic phenotype, 1 in 200 643. All other variants reported here are commonly associated with later onset or variable penetrance with a prevalence of 1 in 5732. The c.644A>G (p.N215S) variant alone has a prevalence of 1 in 11 147.

## Clinical characteristics

One male individual with c.902G>A (p.R301Q), one male individual with the c.644A>G (p.N215S) variant and one female individual with c.718_719del (p.K240fs) variants had HES codes compatible with a known diagnosis of Fabry disease. The remaining participants did not have evidence of an existing diagnosis of Fabry disease in either HES or primary care data. No individuals were identified with a HES or primary care code of Fabry disease who did not have a pathogenic variant.

Age at cohort entry did not differ by variant status at 53.1±8.7 and 55.3±8.4 years vs 56.7±8.2 and 56.3±8.0 years for men and women with and without variants, respectively (p=0.13 and p=0.60, two-sample t-test with unequal variance) (see table 2). The sex split in those with and without Fabry disease-causing variants was not different at 58.3% female and 41.7% in those with variants compared with 55.1% female and 44.9% in the remainder of the biobank cohort (p=0.69, Pearson’s χ^2^). Kidney function was not different for men with Fabry disease-causing variants compared with those without with CKD-EPI eGFRs of 93±14 vs 92±13 mL/min/1.73 m^2^ (p=0.91, two-sample t-test with unequal variance), nor for women 89±15 vs 91±13 mL/min/1.73 m^2^, respectively (p=0.50, two-sample t-test with unequal variance).

The likelihood of microalbuminuria (ACR >3.0 mg/mmol) was higher in men with Fabry disease-causing pathogenic variants (5 of 14) than for the background population (4935 of 82 865, OR 9.33 (95% CI 2.45 to 31.01), p=0.0007), but not for women (2 of 19 vs 5185 of 101 593). Participants with Fabry disease-causing variants were more likely to have a documented family history of cardiovascular disease in HES or reported at the assessment centre than the biobank cohort as a whole (13.9% vs 4.7%, OR 3.31 (95% CI 1.004 to 8.59), p=0.025). For participants with Fabry disease-causing variants, the prevalence of cardiovascular disease was 27.8% compared with 15.0% for the overall biobank cohort (OR 2.19 (95% CI 0.94 to 4.69), p=0.056, Fisher’s exact test). When split by sex, 40% of men had a history of cardiovascular disease compared with 20% for the overall biobank cohort (OR 2.60 (95% CI 0.76 to 8.19), p=0.099), and 19% of women had a history of cardiovascular disease compared with 10% for the overall biobank cohort (OR 2.01 (95% CI 0.49 to 6.17)). There were no differences in the prevalence of either medically recognised or self-reported hearing loss. Acroparasthesia, angiokeratoma, hypohidrosis and corneal verticillata are phenotypic features highly suggestive of classic Fabry disease though do occur in some individuals with late-onset variants.^18^ These manifestations were not noted in our cohort in either HES or primary care records.

Considering the c.644A>G (p.N215S) variant in isolation, one individual had a known diagnosis of Fabry disease and characteristic phenotypic features. Three individuals had cardiac pathology (two atherosclerotic heart disease, one aortic stenosis) which may have been related to Fabry disease, but also had presence of traditional cardiovascular risk factors (hypertension and type 2 diabetes). One woman had microalbuminuria in the absence of other relevant pathology. The remaining 14 individuals with the N215S variant did not have comorbidity suggestive of clinically evident Fabry disease.

The c.1067G>A (p.R356Q) variant has been associated with classic Fabry disease in some published cases.^19^ None of the four individuals (three women, one man) with this variant has a diagnosis of Fabry disease nor clinical features suggestive of Fabry disease.

## Risk cohorts

Using the first phenotype-generated high-risk cohort, we identified 470 individuals meeting the criteria. From the second risk cohort, 488 individuals with exome sequencing data were identified meeting the criteria. Only the individual with a pre-existing diagnosis of Fabry disease with the c.902G>A (R301Q) variant was identified through this approach and was captured in both risk cohorts.

## Discussion

We have shown that the overall prevalence of Fabry disease-causing variants in the UK Biobank is 1 in 5573 with the majority being those associated with a late-onset phenotype. This is similar to and consistent with the 1 in 4600 identified following initial screening by enzyme activity in dried blood spot tests in males in Spada *et al*’s work.^5^ Our findings are also consistent with the figure of 1 in 11 854 from Sawada *et al*’s cohort once the reduced sensitivity of blood spot testing in women is taken into account. It further reinforces the lack of sensitivity of dried blood spot tests in women.^20^

The c.644A>G (p.N215S) variant alone has a prevalence of 1 in 11 147. Evidence for pathogenicity of this variant comes from multiple cohort studies and is associated with later-onset predominantly cardiac manifestations.^18 21^ There was considerable heterogeneity in both severity and age of onset of clinically apparent disease in these studies. Our findings support this observation of heterogeneity and highlights that ascertainment impacts the phenotype associated with a variant. We found variation in the clinical phenotype of the individuals with this variant as ascertained from general practitioner (GP) records, self-report and ICD-10 (International Classification of Diseases version-10) codes. It is possible that this reflects poor sensitivity for finding Fabry disease’s features when using HES data and GP records, alternatively this may reflect variable penetrance. This variant is an example of why the relatively high frequency of Fabry disease’s variants we found, compared with studies based on clinical features of Fabry disease, may include individuals with mild Fabry disease that would have not previously been diagnosed and pathogenic variants with incomplete penetrance. Further evidence for this could be found in future follow-up studies of individuals identified with Fabry disease’s variants in screening studies, and in other population biobanks.

We attempted to identify people who were likely to have Fabry-causing pathogenic variants by identifying individuals with an aggregation of comorbidity which would be highly suggestive of underlying Fabry disease. This approach failed to identify any individuals who did not have a prior diagnosis of Fabry disease who did have a likely pathogenic variant. This finding mirrors the difficulties in identifying individuals with Fabry disease from single clinical characteristics.^10–12^ It is possible that different criteria would provide greater sensitivity. Our results suggest it will be difficult to apply screening criteria to either hospital or GP records with enough sensitivity or specificity to find undiagnosed patients with Fabry disease. It is possible that more detailed analysis or hospital or GP records, or integration of other imaging or biomarker data may in the future be able to. This further highlights the need to develop robust strategies for the identification of people with Fabry disease from clinical cohorts.

One key strength of the current work is that by using exome sequencing, at-risk females will be identified. Like other X-linked conditions, Fabry is under-recognised in females because of lack of sensitivity of existing clinical tests, and diagnosis is more likely to occur when there is an affected male family member.^22^ Newborn screening studies typically use dried blood spot testing for α-Gal A activity,^5–7^ but this is less sensitive in females^20^ and therefore birth studies using this method are likely to underestimate frequency of Fabry disease’s variants in a population.

The majority of participants in whom we identified Fabry-causing variants had neither a known diagnosis of Fabry disease nor pathology highly suggestive of underlying Fabry disease. There are several possible explanations for this: 58% of individuals identified with a Fabry disease-causing pathogenic variant are female in this cohort and females are more likely to have delays or missed diagnoses of Fabry where it is present.^22^ It is also likely that females will have a milder or later-onset phenotype for a given pathogenic variant,^2^ although we were not able to test for differences in phenotype between males and females due to the number of variants we identified in this study. Additionally, the age of UK Biobank participants is younger than the typical age at first presentation with a Fabry indicator presentation, particularly for the variants associated with late onset, that make up the majority of variants we found.^18^ Primary care data are available for only half of the cohort, thus some individuals with pathology attributable to Fabry disease may not be identified. While we have used ACMG/AMP guidelines for classification of pathogenicity of variants,^17^ it remains possible that one or more of our variants are not truly pathogenic.

Previous analyses of UK Biobank data have shown that disease penetrance from a given pathogenic variant may be substantially lower than estimated from studies of cohorts with a known genetic diagnosis.^23^ A key strength of the current work compared with newborn screening studies is that our cohort has had a median of 54 years in which to develop a Fabry-associated phenotype. From this, we see that the majority of those who have a late-onset associated variant will not develop Fabry-associated pathology by this time. Nevertheless, the difference in ACR in males, family history of cardiovascular disease and numerical differences in cardiovascular disease compared with the background biobank cohort suggests the possibility of undiagnosed Fabry disease in some of our individuals with variants.

The variants identified here were predominantly those which are known to be later-onset variants or for which there is conflicting evidence supporting classification as classic or late onset. Participants in the UK Biobank were between 40 and 69 years of age at entry into the cohort.^14^ The age of participants favours later-onset variants and less severe phenotypes as individuals with classic disease-causing variants being less likely to survive to an age which would have permitted study entry. It is also worth noting that UK Biobank participants have a lower prevalence of cardiovascular disease, renal disease and a longer life expectancy than the background population.^24^ As such, those with disease manifestations from Fabry disease including those with later-onset variants may have been less likely to enter the study. If this were the case, it would only be likely to result in an underestimation of Fabry disease’s variant frequency, and therefore our estimate of frequency could be considered conservatively.

With the difference in the prevalence of family history of cardiovascular disease between those with and without Fabry disease-causing variants, the possibility of clinically evident Fabry disease in the families of the individuals identified here with Fabry disease-causing variants must be considered. Similarly, the higher likelihood of microalbuminuria reported here in men may be an early marker of renal involvement in Fabry disease.

Earlier work has produced conflicting assessments of the phenotypes associated with some variants in terms of late-onset or classic disease. The c.1067G>A (p.R356Q) variant was associated with the classic phenotype in the finding of an Italian case study.^19^ With a reported α-Gal A enzyme activity of 15% of wild type^25^ and the absence of confirmed Fabry or Fabry phenotype in the present work, this would favour classification as a late-onset or reduced penetrance phenotype.

The principle limitation to the current work is that participants in the UK Biobank cannot be recontacted for further study. Consequently, it is not possible to confirm a diagnosis of Fabry disease with measurement of enzyme activity or relevant substrates. Our findings may not be generalisable outside the populations of predominantly European descent. The c.644A>G (p.N215S) variant is known to be the most common late-onset variant in populations of European descent, though it has been described in people of Japanese and Chinese descent.^6 26^ In addition, we cannot exclude the presence of intronic variants such as the c.936+919G>A variant, which has been frequently found in Asian populations.^8^

The optimum method for screening for Fabry disease at population level is not known. Screening based on clinical characteristics has previously been shown to have a low diagnostic yield.^10–12^ We were unable to identify new cases by such an approach in the UK Biobank cohort. Genetic screening of populations such as presented here may identify people with Fabry disease-causing pathogenic variants who do not go on to develop the disease. In the work by Spada *et al* and Sawada *et al*, enzyme activity was assayed on a dried blood spot test before confirmatory genetic testing.^5 6^ While this may increase the likelihood that those identified will develop the disease, it is of limited utility for females.

The clinical consequences of Fabry disease can be devastating with substantial reductions in quality of life and life expectancy as a result of major cardiovascular events, neurological involvement and kidney disease. With the interval between identification of those at risk and onset of clinical manifestations, where they occur, typically being several decades later, significant questions remain around who to screen and when to treat to avoid these consequences.

Fabry disease-causing *GLA* variants are far more prevalent in an unselected population sample than would be expected from the reported prevalence of Fabry disease. This may be because some of the reported variants have reduced pathogenicity or penetrance, or are later-onset causing variants with variable penetrance. Our work suggests that the true prevalence of later-onset Fabry disease is substantially higher than current estimates. As genetic screening becomes more widely used, it will be essential to understand the penetrance of these variants to inform clinical monitoring strategies and identify those who will benefit from treatment.

## Data Availability

Data may be obtained from a third party and are not publicly available. Data are available through the UK Biobank via https://www.ukbiobank.ac.uk/enable-your-research/apply-for-access.
