# Supplementary material for: Prevalence of Fabry disease-causing variants in the UK Biobank
Source: J Med Genet. 2022 Aug 17;60(4):391–6. doi: 10.1136/jmg-2022-108523 (PMC10086508; doi:10.1136/jmg-2022-108523)
Supplement: Supplementary data [file jmg-2022-108523supp001.pdf]

Prevalence and penetrance of Fabry Disease causing variants in the UK population

Primary care data search

Acroparasthesia

Angiokeratoma

Sensorineural hearing loss

Hypohidrosis

Anhydrosis

LVH (ventricular)

Congestive heart failure

Cardiomypathy

Dysrhythmia

Conduction

Stroke

Cerebrovascular

TIA (Transient Ischaemic Attack)

Neuropathy

Ischaemic

The terms above were searched for. Where a word is underlined, the underlined section was searched for in addition to the whole phrase.

Fabry phenotype risk cohorts:

Risk Cohort 1

ICD 10 codes

H903 H904 H905 H906 H907 H908 H912 H918 H919 H933 H938 H939 I200 I201 I208 I209 I210 I211 I212 I213 I214 I219 I248 I249 I250 I251 I252 I255 I256 I258 I259 I250 I251 I252 I255 I256 I258 I259 I421 I422 I425 I428 I429 I431 I438 I440 I441 I442 I443 I444 I446 I447

I450 I451 I452 I453 I454 I455 I456 I458 I459 I460 I461 I469 I460 I461 I469 I480 I481 I482 I483 I484 I489 I490 I491 I492 I493 I494 I498 I499 I500 I501 I509 I517 I518 I519

ICD 9 codes

3891 3892 3899 4109 4119 4129 4139 4140 4148 4149 4254 4260 4263 4264 4266 4267 4269 4271 4272 4273 4274 4275 4278 4279 4280 4281 4292

Risk Cohort 2

ICD 10 codes

I630 I631 I632 I633 I634 I635 I636 I638 I639 I64 I660 I661 I662 I663 I664 I668 I669 I672 I678 I679 I200 I201 I208 I209 I210 I211 I212 I213 I214 I219 I248 I249 I250 I251 I252 I255 I256 I258 I259 I250 I251 I252 I255 I256 I258 I259 I421 I422 I425 I428 I429 I431 I438 I440 I441 I442 I443 I444 I446 I447 I450 I451 I452 I453 I454 I455 I456 I458 I459 I460 I461 I469 I460 I461 I469 I480 I481 I482 I483 I484 I489 I490 I491 I492 I493 I494 I498 I499 I500 I501 I509 I517 I518 I519

ICD 9 Codes

4109 4119 4129 4139 4140 4148 4149 4254 4260 4263 4264 4266 4267 4269 4271 4272 4273 4274 4275 4278 4279 4280 4281 4292 4349 4359 4369 4371 4379
