# Supplementary material for: Prevalence of Fabry disease-causing variants in the UK Biobank
Source: J Med Genet. 2022 Aug 17;60(4):391–6. doi: 10.1136/jmg-2022-108523 (PMC10086508; doi:10.1136/jmg-2022-108523)
Supplement: Supplementary data [file jmg-2022-108523supp002.pdf]

	N215S		Non-N215S	
	Male	Female	Male	Female
n(%)	5(14)	13(36)	10(28)	8(22)
Age (years), mean(SD)	52.4±11	55.3±8	53.5±7.8	55.25±9.6
Diagnosed Fabry Disease	1	0	1	1
Cardiovascular disease, n(%)	2	3	4	1
Hearing impairment, n(%)	0	0	0	1
CKD-EPI eGFR mls/min/1.73m^2 *	98±12	91±12	90±15	85±18
Microalbuminuria #	2	3	3	0

Supplementary table 1: Phenotype data when analysed by presence of N215S data versus non-N215S variants. (*creatinine missing for 1 female, #ACR missing for 1 male).
